# In Vitro and In Silico Studies on Angiotensin I-Converting Enzyme Inhibitory Peptides Found in Hydrophobic Domains of Porcine Elastin

**DOI:** 10.3390/molecules28083337

**Published:** 2023-04-10

**Authors:** Toshiya Hatakenaka, Tamaki Kato, Kouji Okamoto

**Affiliations:** 1 Graduate School of Life Science and Systems Engineering, Kyushu Institute of Technology, Kitakyushu 808-0196, Japan; p899016t@mail.kyutech.jp; 2 Vital Resources Applied Laboratory, Inc., Iizuka 820-0067, Japan

**Keywords:** angiotensin I-converting enzyme inhibition, antihypertension, elastin, molecular docking

## Abstract

One of the most striking aspects of the primary structure in the hydrophobic domains of the tropoelastin molecule is the occurrence of the VAPGVG repeating sequence. Since the N-terminal tripeptide VAP of VAPGVG showed a potent ACE inhibitory activity, the ACE inhibitory activity of various derivatives of VAP was examined in vitro. The results showed that VAP derivative peptides VLP, VGP, VSP, GAP, LSP, and TRP exhibited potent ACE inhibitory activities, while the non-derivative peptide APG showed only weak activity. In in silico studies, the docking score S value showed that VAP derivative peptides VLP, VGP, VSP, LSP, and TRP had stronger docking interactions than APG. Molecular docking in the ACE active pocket showed that TRP, the most potent ACE inhibitory peptide among the VAP derivatives, had a larger number of interactions with ACE residues in comparison with APG and that the TRP molecule appeared to spread widely in the ACE pocket, while the APG molecule appeared to spread closely. Differences in molecular spread may be a reason why TRP exhibits more potent ACE inhibitory activity than APG. The results suggest that the number and strength of interactions between the peptide and ACE are important for the ACE- inhibitory potency of the peptide.

## 1. Introduction

Hypertension is a leading cause of 18 million cardiovascular deaths annually [1]. Thus, hypertension is considered to be one of the risk factors for cardiovascular disease. Angiotensin I-converting enzyme [EC3.4.15.1] (ACE) converts angiotensin I to angiotensin II, a hypertensive factor [2,3]. Inhibition of ACE is therefore anticipated as an important target for lowering blood pressure. Drugs such as captopril, lisinopril, and enalapril [4] are commercially available, effective antihypertensive treatments. However, these drugs are all synthetic and have various side effects, such as allergic reactions, cough, skin rashes, and taste disturbances. Consequently, there is a great demand for ACE inhibitors derived from naturally occurring materials. Thus far, ACE inhibitory peptides derived from many foods and organisms, including animal proteins [2,3,5,6,7,8], marine organisms [9,10,11,12,13,14], plants [15,16,17,18,19,20,21], and insects [22,23], have been studied.

Elastin is the core protein of elastic fiber that provides elasticity and resilience to elastic tissues such as vascular walls, skin, ligaments, and lungs in a variety of vertebrates. Elastin also plays a role in modulating various aspects of cellular function, such as cell migration [24]. The ACE inhibitory peptides VGHyp, VVPG, and VYPGG have been isolated from thermolysin-treated bovine neck ligament elastin [25]. The ACE inhibitory peptides VYPG, VGVAPG, and GYPI have also been isolated from elastase-degraded pig aortic elastin [26]. However, ACE inhibition by these peptides was shown to be less active. Two major types of domains are found in tropoelastin, a precursor of elastin: (1) hydrophobic domains rich in Gly, Val, Pro, and Ala residues, often occurring in repeats of peptides such as VPGVG and VAPGVG; and (2) hydrophilic cross-linking domains rich in Ala and Lys residues, often occurring in repeats of Ala residue.

In our previous study [27], we sought to isolate ACE inhibitory peptides in hydrophilic cross-linking domains from elastase-treated porcine elastin. As a result, the weak or potent ACE inhibitory peptides, AAA, LAA, SAA, QAA, GAA, PAA, and DAA, with IC_50_ of 214, 6.1, 162, 255, 258, 310, and 705 μM, respectively, were found. In this study, a further search for detecting the more potent ACE inhibitory peptides in hydrophobic domains of porcine tropoelastin was carried out in vitro and in silico. In the in vitro study, the potent ACE inhibitory peptides, VLP, VGP, VSP, GAP, LSP, and TRP, and a fairly weak ACE inhibitory peptide, APG, were found. In the in silico study, docking score S values were calculated to evaluate the interaction of ACE inhibitory peptides to ACE, and molecular docking simulation was carried out to clarify the difference between ACE-TRP and ACE-APG interactions.

## 2. Results

### 2.1. Purity of Synthetic Peptides

The purities and molecular weights of newly synthesized peptides were confirmed by high-performance liquid chromatography (HPLC) and mass spectrometry, respectively, and these physicochemical properties are summarized in Table 1. The purities of all peptides were assessed to be more than 90%. The calculated values of the molecular weight of all peptides were in good agreement with the found values.

### 2.2. ACE Inhibitory Activity of Peptides Found in Tropoelastin

The most prominent aspect in hydrophobic domains of the tropoelastin molecule is the occurrence of the repeating VAPGVG sequence. As the ACE inhibitory activity of VAPGVG was fairly weak and its IC_50_ value was 906 μM, the ACE inhibitory activities of VAP at the N-terminal side and GVG at the C-terminal side of VAPGVG were examined and shown to have IC_50_ values of 1.0 and 369 μM, respectively. From the fact that VAP exhibited potent ACE inhibitory activity, it was implied that the N-terminal tripeptide sequence of VAPGVG is involved in ACE inhibition. Hence, VAP was regarded as a parent peptide, and the ACE inhibitory activities of five peptides, VA, AP, GAP, VSP, and TRP, which are derivatives of VAP, and two peptides, PG and APG, which are not derivatives of VAP, were examined, and the results are shown in Table 2. Five peptides, which are derivatives of VAP, are all contained in the hydrophobic domains of the tropoelastin molecule, as shown in Table 3. The ACE inhibitory activities of three peptides, LSP [28], VGP [29], and VLP [29], reported by Miyoshi et al. [28] and Wu et al. [29]. are shown in Table 2. These peptides are regarded as derivatives of VAP (Table 2) and are also contained in hydrophobic domains of the tropoelastin molecule (Table 3). The dipeptide VA or AP in which the C-terminal Pro residue or the N-terminal Val residue of VAP is deleted showed weak ACE inhibitory activity, with an IC_50_ value of 326 or 230 μM, respectively. The dipeptide PG and tripeptide APG, which are not derivatives of VAP, exhibited fairly weak ACE inhibitory activities, with IC_50_ values of 1814 and 1194 μM, respectively. The tripeptide VLP, VGP, or VSP, which is an Ala residue of VAP, is substituted by a Leu, Gly, or Ser residue, respectively; the tripeptide GAP, which is a Val residue of VAP, is substituted by a Gly residue; and the tripeptide LSP or TRP, which are Val and Ala residues of VAP, are substituted by Leu and Ser residues or Thr and Arg residues, respectively; and all of these exhibited potent ACE inhibitory activities, with IC_50_ values of 3.9, 26.3, 10.4, 35.0, 1.7, and 1.3 μM, respectively. However, the tripeptide GLG, which is the Val residue of the weak ACE inhibitory peptide GVG, is substituted by a Leu residue and did not show potent ACE inhibitory activity, the same as GVG.

These results proved that the ACE inhibitory activities of peptides that are derivatives of VAP showed higher ACE inhibitory activities than peptides that are not derivatives of VAP. Furthermore, it was demonstrated that the peptides VAP, TRP, LSP, and VLP containing a Pro residue at the C terminus possess potent ACE inhibitory activities, suggesting that the C-terminal Pro residue of VAP is important for ACE inhibition but the Val or Ala residue of VAP is not.

### 2.3. Relationship between the IC_50_ and Docking Score S of ACE Inhibitory Peptides

Docking score S for the docking of ACE inhibitory peptide to ACE (PDB ID code: 1O8A) was calculated using MOE ^®^ 2018, and a value (kcal/mol) of the lowest energy level at the docking state of each peptide and ACE is expressed in Table 2. The calculated docking score S value evaluates the interaction of the ACE inhibitory peptide with the amino acid residues and metal atom Zn(II) in the ACE active pocket and shows that the lower the S value, the stronger the docking interaction. Among the peptides shown in Table 2, the potent ACE inhibitory peptides such as VAP, TRP, LSP, and VLP, with small IC_50_ values of 1.0, 1.3, 1.7, and 3.9 μM, respectively, exhibited low docking score S values of -10.3512, −11.1199, −10.2541, and −10.9502 kcal/mol, which are less than −10 kcal/mol, respectively. However, the weak ACE inhibitory peptides such as VA, AP, PG, APG, GVG, and GLG, with high IC_50_ values of 326, 230, 1814, 1194, 369, and 485 μM, respectively, gave high docking score S values of −9.1838, −8.5065, −8.9506, −9.3226, −9.0309, and −9.4905 kcal/mol, which are more than −10 kcal/mol, respectively.

**Figure 1:**
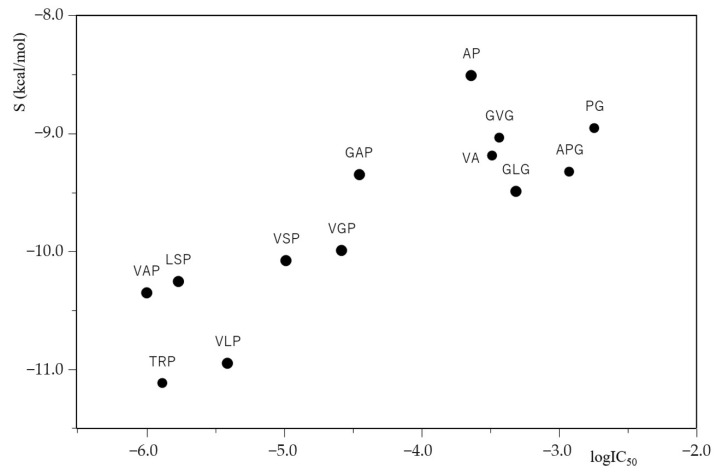
A plot of logIC_50_ of ACE inhibitory peptides against docking score S between ACE inhibitory peptides and ACE (PDB ID code: 1O8A).

The relationship between the common logarithm of IC_50_ (logIC_50_) and the docking score S for ACE inhibitory peptides is plotted in Figure 1. The results show that the smaller the logIC_50_ values of ACE inhibitory peptides, the lower the docking score S values between ACE and its inhibitory peptides, and that the ACE inhibitory activity of peptides with both smaller logIC_50_ values and lower docking score S values were potent. These results suggest that the extent of ACE-inhibiting peptide potency is related to the extent of the tightness of ACE–peptide binding. Furthermore, it is of note that the logIC_50_ values of ACE inhibitory peptides obtained in vitro appear to correlate with the docking score S values of ACE inhibitory peptides obtained in silico.

### 2.4. Informative Data by Online Tools on ACE Inhibitory Peptides

The toxicity, net charge at pH 8.3 and pI, and PepSite2 *p* value for ACE inhibitory peptides, and the number and starting positions of these peptides present in porcine tropoelastin obtained using online tools, are summarized in Table 3. No toxicity was predicted for all peptides. This is an important issue for utilizing these peptides as natural functional food ingredients with ACE inhibitory function. As for the number of peptides present in porcine tropoelastin, GVG was present in large numbers 39, but most of the others had single digit numbers and TRP had just one. In regard to net charge at pH 8.3 and pI, only TRP had a net charge of 0.3 and a pI of 10.00, differing from the others with a net charge of −0.7 and a pI of 5.50. This is probably because TRP contains arginine, a basic amino acid, while the other peptides are composed of neutral amino acids. PepSite2 *p* value predicts how well each peptide binds to ACE (PDB ID code: 1O8A), and a statistically significant *p* value of less than 0.05 predicts that its binding is favorable [8,30]. As shown in Table 3, since the PepSite2 *p* values of all ACE inhibitory peptides were less than 0.05, the binding of these peptides to ACE was predicted to be desirable for ACE inhibition.

### 2.5. Molecular Docking Simulation on ACE Inhibitory Peptides

**Figure 2:**
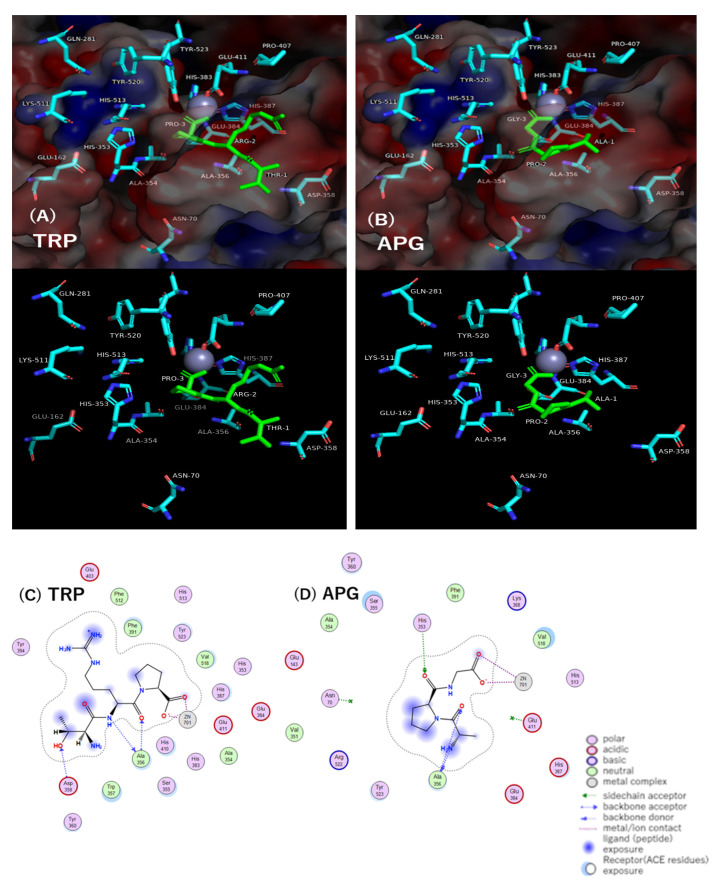
Docking simulation of the interaction between peptides and amino acid residues and Zn(II)701 at active site of ACE (PDB ID code: 1O8A) in the states with the smallest docking score S values of −11.1199 and −9.3226 for TRP and APG, respectively. (**A**,**B**) 3D structure of the complex with peptides and ACE residues. Peptide is shown in green. Red: negative charge of ACE residues, blue: positive charge of ACE residues. The surface charge of ACE is shown in the upper figure and hidden in the lower figure. (**C**,**D**) 2D diagram of intermolecular interaction between peptides and ACE residues.

The interactions between ACE residues and the potent ACE inhibitory peptide TRP and between ACE residues and the fairly weak ACE inhibitory peptide APG in the active pocket of the ACE model (PDB ID code: 1O8A) were investigated in detail by molecular docking simulation using MOE^®^ 2018. The results in the states of four lower docking score S levels and in the state of lowest S level are summarized in Table 4 and depicted in Figure 2, respectively. The TRP with the lowest docking score S value of −11.1199 kcal/mol formed three hydrogen bond interactions with Ala356 and Asp358 of ACE residues and four metal/ion contacts with Zn(II)701 in the ACE active pocket. In more detail, all three hydrogen bond interactions were found between the backbone amide NH of Ala356 and the backbone carbonyl CO of the Arg residue of TRP, between the backbone carbonyl CO of Ala356 and the backbone amide NH of the Arg residue, and between the backbone amide NH of Asp358 and the side-chain hydroxyl group OH of the Thr residue. ACE is a zinc metalloprotease, and three amino acid residues, His387, His383, and Glu411, are coordinated with Zn(II) [31]. Zn(II)701, an important catalytic component, formed four metal/ion contacts with the two oxygen atoms of the C-terminal carboxyl group of Pro. The APG with the lowest docking score S value of −9.3226 kcal/mol formed three hydrogen bond interactions with the Ala356 and His353 of the ACE residues and four metal/ion contacts with Zn(II)701 in the ACE active pocket. In more detail, all three hydrogen bond interactions were found between the backbone carbonyl CO of Ala356 and the N-terminal amino group NH2 of the Ala residue of APG, between the backbone amide NH of Ala356 and the backbone carbonyl CO of the Ala residue, and between the nitrogen atom of the side-chain imidazole group of His353 and the backbone carbonyl CO of the Pro residue. Moreover, four metal/ion contacts were found between Zn(II)701 and the two oxygen atoms of the C-terminal carboxyl group of the Gly residue. In the state of the lowest docking score S level, the number of interactions between ACE and TRP was similar to that between ACE and APG. However, TRP formed 37 interactions with ACE residues in the states of four lower docking score S levels (i.e., −11.1199, −11.0033, −10.8337 and −10.7977 kcal/mol): 12 hydrogen bond interactions, 9 ionic bond interactions, and 16 metal/ion contacts, while APG formed 22 interactions with ACE residues in the states of four lower S levels (i.e., −9.3226, −9.2340, −9.1327 and −9.1070 kcal/mol): 7 hydrogen bond interactions and 15 metal/ion contacts. This demonstrated that a large number of interactions between the peptide and ACE are important for ACE inhibitory potency of the peptide.

The differences in docking score S values between states No.1 and No.2, between states No.1 and No.3, and between states No.1 and No.4 were 0.1166, 0.2862, and 0.3222 kcal/mol for TRP, respectively, and 0.0886, 0.1899, and 0.2156 kcal/mol for APG, respectively, indicating that the values between S levels for APG are smaller than those for TRP. This suggests that APG has an easier transition from the lowest S level to the higher one than TRP does. As shown in Table 4, this easier transition to a higher S level of APG with little conformational change might be a reason why APG exhibits weaker ACE inhibitory activity than TRP does. Furthermore, the TRP molecule in the states of the four lower docking score S levels appears to spread widely through 37 interactions in the ACE active pocket, while the APG molecule in the states of the four lower docking score S levels appears to spread closely through 22 interactions in the ACE active pocket. This wide spread in the ACE active pocket could be one of the reasons why TRP exhibits higher ACE inhibitory activity than APG does. As one of the reasons for the potent ACE inhibition, the importance of a peptide that contains a bulky side group such as Arg has been mentioned by Yathisha et al. [32]. In Table 4, TRP was found to interact with five residues, Ala356, Asp358, Pro407, Glu411, and His387, in the ACE active pocket, while APG was found to interact with three residues, Ala356, Asn70, and His353. The alanine-scan calculation by Fang et al. [33]. suggested that the Pro407 mutation among ACE residues had the greatest influence on the reduction of the stability of the ACE molecule. Since TRP only interacts with Pro407, this could be a reason why TRP has potent ACE inhibitory activity.

## 3. Discussion

In this study, we investigated the ACE inhibitory activity of tropoelastin-derived peptides and made several important findings. First, peptides derived from the tripeptide VAP at the N-terminus of the tropoelastin repeat sequence VAPGVG showed high ACE inhibitory activity, and the presence of a Pro residue at the C-terminus was suggested to be involved in ACE inhibition. Secondly, the correlation between logIC_50_ values and docking score S values indicated that the inhibitory potency of ACE inhibitory peptides is related to ACE-peptide binding, suggesting that molecular docking simulation can be a predictive tool for ACE inhibitory peptides. The results of the molecular docking simulations also showed that the tripeptide TRP, which has strong ACE inhibitory activity, forms more interactions with ACE residues than the tripeptide APG, which has weak ACE inhibitory activity, especially with the important Pro407 residue. Furthermore, the tripeptide TRP is more widely distributed in the active pocket of ACE than the tripeptide APG, which contributes to its higher inhibitory activity. Currently, both molecular dynamics simulations [33] and molecular docking [8,12,21] are useful methods for understanding the formation of enzyme–inhibitor complexes, with the former providing detailed information on enzyme–inhibitor interactions and the latter predicting the steric structure of enzyme–inhibitor complexes. The importance of molecular dynamics simulations is increasing as research progresses from dietary supplements to pharmaceuticals. Although molecular docking provides rapid, easy-to-understand information for those new to computational science, the insights gained from molecular dynamics simulations are indispensable for drug development, despite the longer computational times required. The integration of both types of simulations can provide a more comprehensive understanding of protein–ligand interactions, potentially accelerating the discovery and optimization of effective drug candidates. Should peptides with enhanced ACE inhibitory activity be identified in the future, the use of both simulations will be critical to the successful development of effective and safe therapies to improve human health and may provide additional insights. In this study, we may have demonstrated the possibility of finding effective inhibitors from natural products using a relatively simple approach.

## 4. Materials and Methods

### 4.1. Peptide Synthesis

Peptides found in the hydrophobic domains of porcine tropoelastin (i.e., 1 hexapeptide VAPGVG; 7 tripeptides VAP, APG, GAP, VSP, TRP, GVG, and GLG; 3 dipeptides VA, AP, and PG) were synthesized by a conventional solid-phase method using the Fmoc(9-fluorenylmethyloxycarbonyl) strategy [34,35,36,37]. The procedure for deprotection of the Fmoc group was the use of a DMF (*N*,*N*-dimethylformamide) solution containing 20% piperidine. The coupling procedure was carried out with 0.45 M HBTU (*N*-[(1*H*-benzotriazole-1-yl)-(dimethylamino)methylene]-*N*-methylmethanaminium hexafluorophosphate *N*-oxide) and HOBt (1-Hydroxybenzotriazole) in DMF solution.

The progress of the coupling reaction and the deprotection of Fmoc was evaluated using a chloranil test kit (Tokyo Kasei Kogyo Co., Ltd., Tokyo, Japan) [38]. HBTU, HOBt, and all amino acid derivatives were purchased from Watanabe Chemical Industries, Ltd. (Hiroshima, Japan). All other organic reagents were purchased from Fujifilm Wako Pure Chemical Corporation (Osaka, Japan). The obtained crude peptides were purified by gel filtration using Sephadex G-10.

### 4.2. Assay of ACE Inhibitory Activity

The ACE inhibitory activity of newly synthesized peptides was measured under the following condition by using Nakano et al.’s method [18]. ACE (20 mU/mL, from rabbit lung) was used for the enzyme solution and 100 mM hippuryl-histidyl-leucine (HHL) was used for the substrate solution. Each solution was prepared in 0.1 M HEPES buffer (pH 8.3, 0.3 M NaCl, 0.01% Triton-X). Both enzyme solution (25 μL) and sample solution (20 μL) were preincubated at 37 °C for 5 min. A total of 100 mM HHL substrate solution (25 μL) was added and incubated at 37 °C for 30 min. The reaction was stopped by the addition of 100 mM NaOH solution (25 μL), followed by the addition of 1% o-phthalaldehyde in methanol (25 μL) and maintained at room temperature for 20 min. Next, 100 mM HCl (25 μL) was added and further maintained at room temperature for 10 min. The amount of His-Leu cleaved from the substrate by ACE was determined by measuring the fluorescence intensity of the product reacted with o-phthalaldehyde (excitation wavelength 370 nm, emission wavelength 460 nm) in a microplate reader. ACE inhibitory activity was calculated by the following formula:

a ACE inhibitory activity (%) = [1 − (a − c)/(b − d)] × 100(1)

where a is the fluorescence intensity in the presence of both ACE and the inhibitor, b is the fluorescence intensity in the presence of ACE without the inhibitor, c is the fluorescence intensity in the presence of the inhibitor without ACE, and d is the fluorescence intensity in the absence of both ACE and the inhibitor. ACE inhibitory activity was expressed by IC_50_ value: the concentration of inhibition required to inhibit 50% of the ACE activity, and each IC_50_ value was measured three times.

### 4.3. Molecular Docking

Molecular docking simulation was performed using the crystal structure of an ACE model imported from the Protein Data Bank (PDB ID code: 1O8A, https://www.rcsb.org/structure/1o8A, accessed on 13 March 2022) [8,12,21,39]. Before docking, all water molecules and unwanted substructures must be removed and polar hydrogen atoms must be added to the ACE model. Optimization of the structure of the ACE model and minimization of the ligand energy was achieved with the help of MOE^®^ 2018 (Molecular Operating Environment, Chemical Computing Group ULC, Montreal, QC, Canada) software using the AMBER10: EHT force field, which optimized hydrogen atom addition. The parameters for Protonate 3D were the same as for ACE inhibitory activity evaluation: temperature 310 K, pH 8.3, and salt 0.3 M. This software was additionally used to score the resulting docking poses of peptides in the ACE active pocket via the London dG and GBVI/WSA dG functions in the software. The peptides used in the simulation were the 10 tripeptides VAP, APG, GAP, VSP, TRP, LSP, VGP, VLP, GVG, and GLG, and the three dipeptides VA, AP, and PG. The following parameters were used: Placement; Triangle Matcher; rescoring 1: London dG; Refinement: Rigid Receptor; and rescoring 2: GBVI/WSA dG [19,40,41]. The 2D interactions of peptides with ACE were shown in MOE^®^ 2018 [42], and the 3D docking poses were displayed in PyMOL 2.0.0a0 [7,20,37,43].

### 4.4. Online Analysis for ACE Inhibitory Peptides

The toxicity, net charge at pH 8.3 and isoelectric point (pI), and PepSite2 *p* values of ACE inhibitory peptides found in porcine tropoelastin were obtained using the online tools ToxinPred [40], PROTEIN CALCULATOR v3.4 [44], and PepSite2 [8,30], respectively. In addition, the number of these peptides and their start locations in the tropoelastin molecule were determined using the following URL (https://www.uniprot.org/uniprotkb/A0A097ZMY9/entry, accessed on 24 March 2022) [45]. ToxinPred (https://webs.iiitd.edu.in/raghava/toxinpred/, accessed on 24 March 2022) was used to predict the toxicity of ACE inhibitory peptides. Protein Calculator v3.4 (http://protcalc.sourceforge.net/, accessed on 24 March 2022) was used to calculate the net charge at pH 8.3 and pI for these peptides. PepSite2 (http://pepsite2.russellab.org, accessed on 24 March 2022) was used to predict the statistical significance of ACE–peptide interaction using the ACE model (PDB ID code: 1O8A).

## 5. Conclusions

In this study, the ACE inhibitory activity of peptides found in the hydrophobic domains of porcine tropoelastin was investigated in vitro and in silico. In vitro study showed that, among the six peptides, VLP, VGP, VSP, GAP, LSP, and TRP, which are derivatives of the potent ACE inhibitory peptide VAP at the N-terminal side of VAPGVG, an occurrence in hydrophobic domains of tropoelastin, TRP exhibited the most potent ACE inhibitory activity, with an IC_50_ value of 1.3 μM. In silico studies suggest that the docking score S value of the most potent inhibitory peptide, TRP, was the lowest among the derivatives of VAP, predicting a strong docking interaction between TRP and ACE. Moreover, molecular docking in the ACE active pocket demonstrated that TRP formed a large number of interactions with ACE residues in the states of four lower docking score S levels, implying that TRP appears to spread widely in the ACE active pocket. It is of note that the ACE inhibitory potency of TRP obtained in vitro appears to correlate with the docking mode of TRP to ACE obtained in silico. The fact that peptides are non-toxic is an important issue for utilizing peptides as natural functional food ingredients with ACE inhibitory function. Although no biochemical toxicity studies of peptides have been conducted, all peptides are predicted to be non-toxic based on data obtained from online tools.

## Figures and Tables

**Table 1:** Physicochemical properties of newly synthesized peptides.

Peptide ^a^	Purity (%) ^b^	MS ^c^ Calcd	MS ^c^ Found
VAPGVG	98.8	498.50	498.80
VAP	99.8	285.34	285.33
VA	95.0	188.23	188.25
AP	95.2	186.21	186.20
PG	98.3	172.18	172.20
APG	95.2	243.26	243.20
GAP	99.0	243.26	243.20
VSP	99.8	301.34	301.20
TRP	90.4	372.42	372.30
GVG	98.9	231.25	231.20
GLG	97.8	245.28	245.20

^a^ All of the peptides are found in hydrophobic domains of porcine tropoelastin. ^b^ Purity was analyzed by HPLC. ^c^ MS means mass spectrometry.

**Table 2:** IC_50_ and docking score S values of ACE inhibitory peptides.

Peptide ^a^	IC_50_ (μM)	S (kcal/mol)
VAP	1.0	−10.3512
VA	326	−9.1838
AP	230	−8.5065
PG	1814	−8.9506
APG	1194	−9.3226
GAP	35.0	−9.3464
VSP	10.4	−10.0776
TRP	1.3	−11.1199
LSP ^b^	1.7 ^d^	−10.2541
VGP ^b^	26.3 ^d^	−9.9914
VLP ^c^	3.9 ^e^	−10.9502
GVG	369	−9.0309
GLG	485	−9.4905

^a^ All of the peptides are found in hydrophobic domains of porcine tropoelastin. ^b^ LSP and VGP are peptides from α-Zein hydrolysate, as reported by Miyoshi et al. [28]. ^c^ VLP is a peptide from the ACE inhibitory peptide database, as reported by Wu et al. [29]. ^d^ These are values reported by Miyoshi et al. [28]. ^e^ This is a value reported by Wu et al. [29]. The values of IC_50_ were measured three times (*n* = 3).

**Table 3:** Informative data on ACE inhibitory peptides obtained using online tools.

Peptide	Toxicity ^a^	The Number of Peptide in Tropoelastin ^b^	Start Location ^b^	Net Charge at pH 8.3 ^c^	pI ^c^	PepSite2 *p* Value ^d^
VAP	Non-Toxin	6	492, 498, 504, 510, 516, 690	−0.7	5.50	0.000028
GAP	Non-Toxin	5	249, 283, 306, 468, 531	−0.7	5.50	0.000063
VSP	Non-Toxin	2	370, 659	−0.7	5.50	0.000130
TRP	Non-Toxin	1	682	0.3	10.00	0.000056
LSP	Non-Toxin	2	421, 698	−0.7	5.50	0.000167
VGP	Non-Toxin	2	173, 620	−0.7	5.50	0.000146
VLP	Non-Toxin	3	133, 146, 255	−0.7	5.50	0.000221
GVG	Non-Toxin	39	40, 89, 92, 110, 122, 143, 172, 230, 258, 260, 263, 313, 318, 323, 328, 333, 338, 343, 348, 353, 358, 363, 390, 392, 400, 410, 412, 466, 495, 501, 507, 513, 562, 571, 604, 619, 635, 638, 643	−0.7	5.50	0.008118
GLG	Non-Toxin	11	21, 24, 48, 52, 86, 95, 485, 641, 646, 674, 709	−0.7	5.50	0.002949

^a^ ToxinPred were used to predict the toxicity of peptides. (https://webs.iiitd.edu.in/raghava/toxinpred/, accessed on 24 March 2022). ^b^ The number of peptides found in hydrophobic domains of porcine tropoelastin and the location of the first residue of the peptide. (https://www.uniprot.org/uniprotkb/A0A097ZMY9/entry, accessed on 24 March 2022). ^c^ PROTEIN CALULATOR v3.4 was used to calculate the net charge at pH 8.3 and the isoelectric point (pI) for the peptides. (http://protcalc.sourceforge.net/, accessed on 24 March 2022). ^d^ *p* value was calculated using PepSite2 to predict the statistical significance of ACE (PDB ID code: 1O8A)-peptide binding. The smaller this value is, the better the binding of the peptide to ACE is predicted. (http://pepsite2.russelllab.org/, accessed on 24 March 2022).

**Table 4:** Intermolecular interaction of TRP or APG with ACE (PDB ID code: 1O8A) in the states of four lower docking score S levels.

Peptide	State	Docking Score S (kcal/mol)	Amino Acid Residues and Zn(II) in the ACE Active Pocket							
			Ala356	Asp358	Pro407	Glu411 *	His387 *	Asn70	His353	Zn(II)701
TRP	1	−11.1199	✓✓	✓						○○○○
	2	−11.0033	✓✓	✓	✓	✓◎				○○○○
	3	−10.8337	✓			◎	◎◎◎◎			○○○○
	4	−10.7977	✓			✓✓◎◎	◎			○○○○
APG	1	−9.3226	✓✓						✓	○○○○
	2	−9.2340	✓					✓		○○○○
	3	−9.1327							✓	○○○○
	4	−9.1070							✓	○○○

The symbols for each interaction are as follows. ✓: Hydrogen bond interaction, ◎: Ionic bond interaction, ○: Metal/ion contact, *: Residue coordinated with Zn(II)701 in the ACE active pocket.

## Data Availability

Not applicable.
